# Summer Abundance and Distribution of Proteorhodopsin Genes in the Western Arctic Ocean

**DOI:** 10.3389/fmicb.2016.01584

**Published:** 2016-10-13

**Authors:** Dominique Boeuf, Raphaël Lami, Emelyne Cunnington, Christian Jeanthon

**Affiliations:** ^1^CNRS, Station Biologique, UMR 7144 Adaptation and Diversité en Milieu MarinRoscoff, France; ^2^Sorbonne Universités – UPMC Université Paris 06, Station Biologique, UMR 7144 Adaptation and Diversité en Milieu MarinRoscoff, France; ^3^CNRS, USR 3579, Laboratoire de Biodiversité et Biotechnologies MicrobiennesBanyuls-sur-Mer, France; ^4^Sorbonne Universités – UPMC Université Paris 06, USR 3579, Observatoire OcéanologiqueBanyuls-sur-Mer, France

**Keywords:** proteorhodopsin, photoheterotrophy, SAR11, Arctic Ocean, Mackenzie River

## Abstract

Proteorhodopsins (PR) are phylogenetically diverse and highly expressed proton pumps in marine bacterial communities. The phylogenetic diversity and *in situ* expression of the main PR groups in polar off-shore, coastal and estuarine waters is poorly known and their abundance has not yet been reported. Here, we show that PR gene sequences of the southern Beaufort Sea including MacKenzie shelf and estuary are mainly affiliated to *Gammaproteobacteria, Alphaproteobacteria*, and *Bacteroidetes*. Substantial overlap (78%) between DNA- and cDNA-based librairies indicated *in situ* PR transcription within a large fraction of PR-containing community. Sets of specific qPCR primers were designed to measure the absolute abundances of the major PR types. Spatial and depth profiles showed that PR-containing bacteria were abundant throughout the photic zone, comprising up to 45% of total bacteria. Although their abundance varied greatly with location and depth, *Alphaproteobacteria* predominated in the PR community in all water masses, with SAR11 as the major PR type. Low nutrient concentrations rather than light were the environmental drivers that best explained the abundance and distribution of arctic PR types. Together, our data suggests that PR-based phototrophy could be the major phototrophic prokaryotic process during the Arctic Ocean summer.

## Introduction

Proteorhodopsins (PRs) are prokaryotic retinal-binding integral membrane proteins that function as light-driven proton pumps. Culture-based experiments suggested their implication in various bacterial physiological functions, including ATP generation (Fuhrman et al., 2008), survival processes in nutrient-depleted conditions (Gómez-Consarnau et al., 2010; Steindler et al., 2011; Akram et al., 2013) or light sensing (Jung et al., 2003). After its initial discovery on a large genomic fragment derived from an uncultured marine gammaproteobacterium of the SAR86 clade (Béjà et al., 2000), PRs have been found in a diversity of bacterial groups notably in the SAR11 clade, one of the most common clades in the oceans (Giovannoni et al., 2005). They have also been identified in the alphaproteobacterial SAR116 clade (Oh et al., 2010; Grote et al., 2011) the gammaproteobacterial clade SAR92 (Stingl et al., 2007b), in *Roseobacter* clade and *Bacteroidetes* species (Gómez-Consarnau et al., 2007; González et al., 2008; Riedel et al., 2010; Yoshizawa et al., 2012) and in marine planktonic *Archaea* (Frigaard et al., 2006). Microbial rhodopsins are widespread and abundant in temperate and tropical marine waters. They have been detected in the Atlantic and Pacific oceans, in the Mediterranean and Red seas (Béjà et al., 2001; Venter et al., 2004; Rusch et al., 2007; Sabehi et al., 2007; Campbell et al., 2008). Some close homologs to PR are found in brackish, freshwater and sea ice environments (Atamna-Ismaeel et al., 2008; Koh et al., 2010).

PR photosystems have been detected in a large percentage of ocean surface-dwelling prokaryotes but estimates vary depending on the oceanic region and detection method. They accounted for up to 65% of total prokaryotes in the Sargasso Sea, 35% in the North Sea and 13% in the Mediterranean Sea, and ranged from 7 to 57% in the North Atlantic Ocean (Venter et al., 2004; Sabehi et al., 2005; Rusch et al., 2007; Campbell et al., 2008; Riedel et al., 2010). However, there have been few quantitative assessments of PR gene abundance in marine environments, especially in high latitudes.

The Arctic Ocean is a largely land-locked basin with a cold, brackish upper mixed layer. The upper photic waters are separated from deeper waters by a strong halocline that is maintained by large river inputs and the annual formation and melting of sea ice (Aagaard et al., 1981). The physical isolation, perennially cold water temperatures, and extreme annual light cycle provide a unique marine habitat for microorganisms (Carmack and MacDonald, 2002). PR gene diversity has been reported in a few arctic coastal sites (Cottrell and Kirchman, 2009; Nguyen et al., 2015) but the abundance of PR-containing bacteria has not been examined at a large scale in a polar marine basin such as the western Arctic Ocean.

We examined the phylogenetic diversity and abundance of PR genes along a 3,000 km transect from the North East Pacific to the Arctic Ocean, which mainly focused on the Beaufort Sea and the Mackenzie shelf and estuary. Our objectives were to identify the major bacterial groups that actively expressed PR in the Arctic Ocean during summer, to assess their absolute abundance and to determine which environmental factors control their distribution. We expected that most PR-containing bacteria actively transcribe the PR gene during arctic summer and that the distribution of major PR types differed in open and coastal oceanic arctic waters in response to different environmental parameters.

## Materials and Methods

### Sampling and Oceanographic Parameters

The MALINA cruise took place onboard the Canadian research icebreaker *CCGS Amundsen* during summer 2009 from Victoria (BC, Canada) to the Beaufort Sea (Leg 1b) and then throughout the Beaufort Sea (Leg 2b) (Supplementary Figure S1). Most of the stations sampled on the west–east transect in the Beaufort Sea (Leg 2b) were ice-free. However, surface waters of eastward offshore waters were still ice-covered. Surface seawater samples were collected with an acid-cleaned bucket during Leg 1b and in the Mackenzie plume (stations 395, 398, 694, and 697) during Leg 2b. In the Beaufort Sea, seawater was collected from six depths (up to 150 m) using Niskin bottles mounted on a CTD (conductivity temperature depth probe) rosette. The methods used to collect the ancillary data of temperature, salinity, pH, dissolved oxygen, colored dissolved organic matter (cDOM), photosynthetically active radiation (PAR), inorganic and organic nutrients, and chlorophyll *a* data are provided in the Supplementary Material. Bacterioplankton biomass for DNA and total RNA extraction was collected onboard as previously described (Boeuf et al., 2013).

### Diversity Analysis of PR Genes and Transcripts

DNA was extracted as previously described (Marie et al., 2006) and concentrated using 100 kDa Amicon Ultra centrifugal filter device (Millipore). Total RNA was extracted using the RNeasy Mini kit (Qiagen) as in Boeuf et al. (2013). ThermoScript RT-PCR system (Invitrogen) was used for the reverse transcription of PR mRNA from total RNA samples. The cDNA synthesis was performed at 55°C using the PR1-aR primer as gene-specific primer (Supplementary Table S1). PR genes were detected by PCR amplification with degenerate primers (Supplementary Table S1) using conditions described by Campbell et al. (2008). Primers pairs from Atamna-Ismaeel et al. (2008) targeting actinorhodopsin-like sequences were also used but did not yield positive PCR products. PR genes and transcript amplicons were cloned directly or after gel extraction using the TOPO4-TA cloning kit (Invitrogen) according to the manufacturer’s instructions. Recombinant clones were sequenced as in Boeuf et al. (2013).

The sequences were trimmed to remove any vector and primer sequences. DNA sequences were translated into amino acid sequences and aligned according to the tertiary structure of proteins using the E-INS-I strategy and BLOSUM62 substitution matrix from MAFFT (Katoh et al., 2009). The resulting protein alignment was back translated to nucleotide acid sequences using Geneious software (Drummond et al., 2012). A conservative value of 82% amino acid sequence similarity (Campbell et al., 2008) was chosen for clustering sequences into operational taxonomic units (OTUs) using MOTHUR (Schloss et al., 2009). Representative sequences (defined as the sequence with the minimum distance to all other sequences in the OTU) were obtained using MOTHUR. The coverage value (Mullins et al., 1995), the Shannon index, *H_Shannon_* (Shannon, 2001) and the bias-corrected richness-estimator, S_Chao1_ (Chao et al., 2005) were calculated for each library (Supplementary Table S2).

A PR gene database containing about 2,000 aligned sequences of cultured species and environmental sequences retrieved from GenBank was constructed using Geneious (Drummond et al., 2012). A phylogenetic tree was constructed with entire protein sequences by Bayesian inference using MrBayes 3.0 (Ronquist and Huelsenbeck, 2003) with mixed models of amino acid substitution estimated by Markov chain Monte Carlo (MCMC) procedure. Four parallel MCMC chains of 1.5 million generations were run and trees were sampled every 100 generations. A consensus tree was constructed after the exclusion of 13,000 ‘burn-in’ trees. The PR gene database and the consensus tree were imported into ARB software (Ludwig et al., 2004). Representative sequences of each OTU and short PR gene environmental sequences were aligned as above and added to the consensus Bayesian tree using the ADD_BY_PARSIMONY algorithm implemented in ARB. Non-informative taxa were removed from the final tree.

### Quantification of PR Gene Abundance

Eight major PR-containing bacterial groups were targeted by qPCR. When primers specific for these clades were not available in literature, they were designed using ARB (Supplementary Table S1). OTU3b (alphaproteobacterial “Arctic” group), OTU1 (alphaproteobacterial HOT2C01 cluster), OTU9 and OTU11 (gammaproteobacterial SAR92 clusters), OTU10 (gammaproteobacterial *Glaciecola* cluster) and a cluster of four *Flavobacterium* arctic isolates were targeted by qPCR to assess their distribution and absolute contribution to the arctic bacterial community. The abundance of SAR11 PR clade and of subcluster OTU3a (alphaproteobacterial “Arctic” group) were assessed by using primers sets designed by Campbell et al. (2008) and Cottrell and Kirchman (2009), respectively. The 16S rRNA genes for all bacteria and for SAR11 clade were quantified using primers (Supplementary Table S1) and conditions described by Suzuki et al. (2000, 2001). Plasmid clones from our Beaufort Sea PR and 16S rRNA libraries were used as positive controls and in standard curves (10-fold dilution series) in the qPCR assays. Plasmid DNA was linearized with *Hind*III and quantified using the Picogreen assay (Invitrogen).

QPCR was performed in triplicate with 1 μl of environmental DNA normalized to 100 pg.μl^-1^ or plasmid DNA solution in a total reaction volume of 10 μl using the ABsolute QPCR SYBR Green/ROX Master Mix kit (Thermo Scientific). QPCR assays were performed using a Chromo4 Real-Time PCR Detection System and the Opticon Monitor software package (Bio-Rad) with the following thermal cycling conditions: 95°C for 15 min, 45 cycles of denaturation at 95°C for 45 s, primer annealing at the primer-specific temperature (Supplementary Table S1) for 45 s with fluorescence measurements after this step, and polymerase extension at 72°C for 15 s. Melting curves were generated after each assay to check the specificity of the amplification by heating from 65 to 95°C at a rate of 0.1°C.s^-1^ and taking fluorescence measurements every 5 s. Only single peaks were observed in the dissociation curves for both the standards and samples, indicating specific amplification with each set of primers. Average amplification efficiencies were as follows: bacterial 16S rDNA = 103.7% (± 0.9), SAR11 16S rDNA = 95.3% (± 0.36), SAR11-PR = 95.6% (± 1.16), summer clade (OTU3a-PR) = 98.3% (± 1.39), OTU3b-PR = 104.3% (± 2.81), OTU11-PR = 98.4% (± 0.98), OTU9-PR = 95.1% (± 2.03), OTU1-PR = 95.9% (± 0.84), OTU10-PR = 101% (± 3.05) and *Flavobacterium*-PR = 103.7%.

No inhibition was detected when a known quantity of standard was added to DNA extracts (data not shown). In order to estimate the fraction of the bacterial community bearing each PR gene type, the estimates of PR gene abundance were normalized to 16S rRNA gene abundance, assuming one PR gene copy and 1.9 16S rRNA copies per genome (Venter et al., 2004; Campbell et al., 2008). The fraction of the SAR11 community bearing PR gene was assessed after normalization of SAR11 PR gene abundance to that of SAR11 16S rRNA gene, assuming one copy of both genes per genome in SAR11 cells. To estimate total abundances of PR-containing bacteria, we summed the abundance estimates of the eight-targeted groups.

### Statistical Analyses

All statistical analyses have been conducted using XLstat software (Addinsoft, France). Links between abundances and environmental variables were explored using Pearson’s correlation tests. The similarity between clone libraries was assessed by a Bray–Curtis distance matrix calculated after resampling and was inferred with the UPGMA algorithm.

### Nucleotide Sequence Accession Numbers

The environmental PR gene sequences obtained in this study are available in GenBank database under the accession numbers JX863105 to JX863220 (DNA sequences) and JX863221 to JX863333 (cDNA sequences).

## Results

### Community Composition and Diversity of PR Sequences

Of a total of 617 PR gene sequences retrieved along the transect covering the North Pacific Ocean to the Beaufort Sea, 306 were obtained from DNA and 311 from cDNA libraries. 72 distinct OTUs were identified after clustering sequences at 82% amino acid sequence similarity (Campbell et al., 2008). Coverage estimates (>80%) and rarefaction curves (data not shown) indicated that most of the diversity was sampled at both offshore and shelf stations (Supplementary Table S2). This observation was confirmed by S_Chao1_ values (estimated numbers of expected OTUs), which agreed with the numbers of retrieved OTUs. The diversity of PR types, assessed with Shannon index, was generally higher in surface than in deeper waters with the highest diversity observed in the Mackenzie River mouth while the coverage index was low (Supplementary Table S2).

**FIGURE 1:**
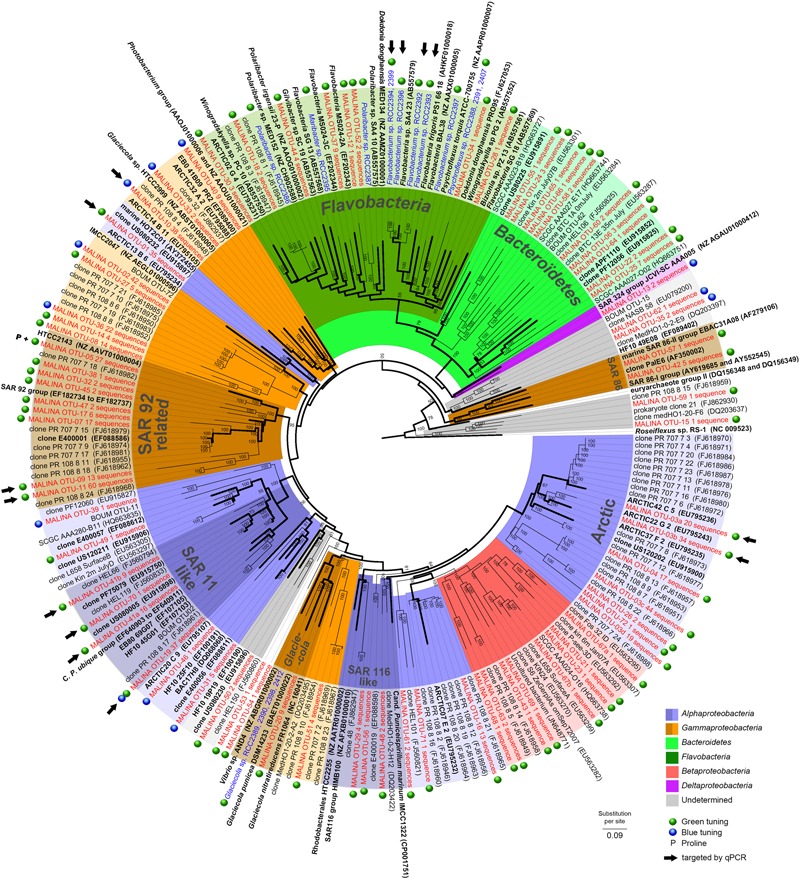
**Phylogenetic tree showing the relationships between PR gene sequences from MALINA environmental samples (in red) and isolates (in blue) (Supplementary Material), and from relatives retrieved in GenBank (in black).** Blue and green dots show the predicted PR absorption of blue or green light as inferred from the amino acid sequence according to Man et al. (2003) (Supplementary text). P indicates PR variants with proline at position 105. Black arrows indicate OTUs targeted by qPCR assays. The tree is based on a bayesian tree to which short sequences were added by ARB_PARSIMONY. Initial bayesian tree is shown in bold lines. The numbers on nodes represent branch confidence values. The scale bar represents dissimilarity between amino acid positions.

A majority of the sequences (>72%, 18 OTUs) were found in both DNA and cDNA libraries. A total of 16% of the sequences were only recovered in DNA libraries (34 OTUs) while 11% were restricted only to cDNA librairies (20 OTUs). A phylogenetic tree was constructed from all 72 OTUs identified in this study and from previous studies (**Figure 1**). Our phylogenetic analysis revealed that most PR sequences were associated with classes *Gammaproteobacteria* (20 OTUs, ∼46% of the sequences) and *Alphaproteobacteria* (16 OTUs, ∼43% of the sequences). Sequences affiliated with the *Bacteroidetes* accounted for an equivalent number of OTUs (18) but a lower proportion of sequences (∼7%). Other OTUs (∼4% of the sequences) were related to classes *Betaproteobacteria or Deltaproteobacteria* or remained unassigned. Hierarchical clustering analysis of the PR community composition clearly separated surface and deep samples (Supplementary Figure S2). *Alphaproteobacteria* generally dominated in surface samples while *Gammproteobacteria* prevailed in deeper layers.

About half of the alphaproteobacterial PR clones (117 sequences) grouped exclusively with PR sequences from the Chukchi, Beaufort, and Bering Seas (Cottrell and Kirchman, 2009). This “Arctic group” contained OTU3a that corresponded to an “Arctic summer clade” revealed by Cottrell and Kirchman (2009). Sequences related to the SAR11 clade including *Candidatus* ‘Pelagibacter ubique’ (91–100% similarity) contributed for 10% of PR clones, most of them retrieved from the RNA library. One of the largest alphaproteobacterial clades contained sequences closely related to the HOT2C01 fosmid gene, a PR type highly abundant the Sargasso Sea (Campbell et al., 2008).

Most gammaproteobacterial clones were closely related to Arctic sequences and belonged to novel OTUs. However, others including OTU9 and OTU11 (13 and 60 sequences) grouped with cultured representatives of the SAR92 group (Cho and Giovannoni, 2004; Stingl et al., 2007b; Oh et al., 2010). OTU10 (38 sequences) showed significant levels of similarity (>79%) with the PR gene of *Glaciecola* sp. HTCC2999. Only a few clones were assigned to the gammaproteobacterial SAR86 group, an abundant uncultivated ubiquitous marine lineage (Sabehi et al., 2004; Dupont et al., 2012). About half of OTUs affiliated to *Bacteroidetes* belonged to the class *Flavobacteria*.

### Abundance and Distribution of Main PR Types

Using the dataset of PR sequences retrieved from the Beaufort Sea, we designed qPCR primers to target six major groups of PR types namely the alphaproteobacterial OTU3b and OTU1, the SAR92-related OTU9 and -11, the *Glaciecola*-related OTU10 and a cluster of 4 isolates related to *Flavobacterium frigoris* (Boeuf, 2013). Primers sets designed by Campbell et al. (2008) and Cottrell and Kirchman (2009) were also used to investigate the abundance of SAR11 PR and OTU3a clades, respectively. The design of universally conserved qPCR primers able to capture most PR types is impossible. Therefore, to estimate total abundances of PR-containing bacteria, we added together the abundance estimates of all eight tested microbial groups containing PR genes (**Table 1**). In the Beaufort Sea (**Table 1**), the total PR relative abundances ranged from below 1 to 45% with a mean contribution of about 15% of total bacteria.

**Table 1:** Mean relative abundances of PR sequence types in the Beaufort Sea as quantified by qPCR (the number of samples analyzed (*n*) is indicated).

Affiliation	PR type	Water masses												All samples		
		River plume surface			Shelf surface			Offshore surface			Other depths					
		(*n* = 4)			(*n* = 19)			(*n* = 8)			(*n* = 42)			(*n* = 73)		
		Mean	*SD*^a^	(Min; Max)	Mean	*SD*	(Min; Max)	Mean	*SD*	(Min; Max)	Mean	*SD*	(Min; Max)	Mean	*SD*	(Min; Max)
SAR11 clade	SAR11	5.4	3.3	(2.2; 9.2)	7.1	6.9	(0; 27.8)	9.9	6.6	(1.6; 21)	12.4	8.9	(0.7; 35.3)	10.4	8.3	(0; 35.3)
Arctic clade	OTU3a	3.4	0.5	(2.8; 3.9)	2.7	3	(0.1; 9.8)	4.8	6.3	(0.5; 18.9)	1.0	0.9	(0; 4.7)	2.5	2.9	(0; 18.9)
Arctic clade	OTU3b	0.8	0.5	(0.3; 1.5)	0.6	0.6	(<0.1; 2.1)	2.9	1.7	(0.7; 5.2)	0.5	0.7	(<0.1; 3.1)	0.8	1.1	(<0.1; 5.2)
HOT2C01-related	OTU1	4.0	1.9	(1.5; 6.1)	1.9	2.2	(0.2; 9.6)	2.0	0.9	(0.8; 3)	2.0	1.6	(0.2; 7.6)	2.1	1.7	(0.2; 9.6)

*Alphaproteobacteria*	Total	13.6	4.7	(6.8; 17.5)	11.8	9.5	(0.6; 34)	19.1	10.9	(6.7; 38.3)	15.3	10.6	(1; 43.9)	14.7	10.2	(0.6; 43.9)

SAR92	OTU9	0.1	0.03	(0.1; 0.2)	0.2	0.3	(0; 1.3)	0.3	0.2	(0.1; 0.6)	0.2	0.2	(<0.1; 0.7)	0.2	0.2	(0; 1.3)
SAR92	OTU11	0.1	0.1	(<0.1; 0.3)	0.2	0.2	(0; 0.8)	0.1	0.1	(<0.1; 0.3)	0.5	0.5	(0; 2.8)	0.4	0.4	(0; 2.8)
HTCC2999-related	OTU10	0.3	0.3	(0.1; 0.7)	<0.1	0.06	(0; 0.2)	0.2	0.1	(<0.1; 0.4)	0.1	0.1	(0; 0.5)	0.1	0.1	(0; 0.7)

*Gammaproteobacteria*	Total	0.6	0.4	(0.2; 1)	0.4	0.3	(<0.1; 1.5)	0.5	0.4	(0.2; 1.2)	0.7	0.6	(0.1; 3.4)	0.6	0.5	(<0.1; 3.4)

*Flavobacteria*	*F. frigoris*	<0.1	0.01	(0;<0.1)	<0.1	0.01	(0;<0.1)	0.1	0.06	(<0.1; 0.2)	<0.1	0.01	(0;<0.1)	<0.1	0.04	(0; 0.2)

All targeted group	Total	14.2	4.9	(7; 17.9)	12.2	9.5	(0.9; 34.4)	19.7	11.1	(7.2; 39.4)	16.0	10.7	(1.1; 44.7)	15.4	10.3	(0.9; 44.7)

*Water masses were delineated as in Boeuf et al. (2013).*

*^a^SD, standard deviation.*

Water categories as defined in Boeuf et al. (2013) using temperature and salinity profiles displayed close mean PR contributions with the highest proportions obtained in offshore waters. Absolute abundances of PR types did not match the relative abundances in clone libraries. Alphaproteobacterial PR types (>95% of the total PR groups) dominated in all water types. Although, weak variations in their mean relative abundance were obtained in the different water categories, distinct biogeographical patterns occurred at the clade level.

The SAR11 PR-containing bacteria prevailed in all water types of the Beaufort Sea and their mean surface contributions doubled from the river plume to offshore waters. Highest surface contributions were recorded near the Amundsen Gulf and in the central Beaufort Sea (Supplementary Figure S3). In most shelf and offshore stations, SAR11 PR abundances increased with depth and peaked (up to 35%) below or above the chlorophyll maximum (Supplementary Figure S4). The highest SAR11 PR contributions (>30%) were obtained offshore at 50 m depth. The second most abundant PR type, OTU 3a, had higher contributions in surface that increased from the river plume to offshore waters (Supplementary Figure S3). The OTU3b PR type showed the same distribution pattern but was less abundant. Both latter clades decreased sharply with depth (Supplementary Figure S4). The HOT2C01-related OTU 1 displayed an opposite distribution pattern, decreasing significantly from the river plume to offshore waters (Supplementary Figure S3). Surprisingly, contributions of gammaproteobacterial PR groups were generally low, ranging from less than 0.1 to 3.4% (Supplementary Figure S3). Their highest contributions were obtained in shelf surface (OTU9) and deeper waters (OTU11). *Flavobacterium* PR type was generally poorly detected in the Beaufort Sea where its highest abundances were obtained in offshore surface samples (Supplementary Figure S3). General trends observed in surface waters of the Beaufort Sea were confirmed along the North Pacific Ocean-Beaufort Sea transect (Supplementary Table S3). However, high relative abundances of OTU3a and *Flavobacterium* were obtained in samples of the Chukchi Sea.

### Assessing the Contribution of the PR Gene in the SAR11 Clade

The SAR11 PR types were the major component (63%) of the PR-containing community in the Beaufort Sea (**Tables 1 and 2**). To better understand this contribution, we quantified the 16S rRNA gene abundance of the SAR11 clade by qPCR (**Table 2**). SAR11 represented about the third (32% ± 21%) of the total bacterial community with contributions twice higher in offshore waters than in coastal samples. In all water categories, about a third (32% ± 17%) of the SAR11 community harbored a PR gene (**Table 2**), but these proportions were highly variable among samples (from <5 to 90%).

**Table 2:** Mean relative abundances of SAR11 clade within the total bacterial community and of SAR11 PR type within the SAR11 and the total PR communities in the Beaufort Sea as quantified by qPCR (the number of samples analyzed (*n*) is indicated; minimum and maximum values are in parentheses).

	Water masses												All samples		
	River plume surface			Shelf surface			Offshore surface			Other depths					
	(*n* = 4)			(*n* = 19)			(*n* = 8)			(*n* = 42)			(*n* = 73)		
	Mean	*SD*^a^	(Min; Max)	Mean	*SD*	(Min; Max)	Mean	*SD*	(Min; Max)	Mean	*SD*	(Min; Max)	Mean	*SD*	(Min; Max)
SAR11 16S rRNA/total bacteria^b^	18.9	11.6	(7.7; 31.4)	19.8	13.3	(3.8; 41.7)	35.4	20.9	(13.2; 79.3)	36.9	21.7	(2.8; 81.6)	31.7	20.9	(2.8; 81.6)
SAR11 PR/SAR11 16S rRNA	28.8	1.8	(26.7; 31.0)	32.2	24.7	(4.4; 91.1)	26.9	8.9	(11.7; 38.2)	32.9	14.6	(9.6; 80.9)	31.8	16.9	(4.4; 91.1)
SAR11 PR/total PR^c^	36.4	13.1	(21.4; 51.5)	50.2	22.8	(21.3; 80.9)	49.2	18.7	(21.6; 76.8)	73.3	13.5	(33.0; 90.2)	63.0	21.9	(21.3; 90.2)

*Water masses were delineated as in Boeuf et al. (2013).*

*^a^SD, standard deviation.*

*^b^SAR11 PR gene copies were normalized to 1.9 16S rRNA gene copies.*

*^c^SAR 11 PR gene copies were normalized to the sum of PR gene copies.*

### Links between Main PR Types and Environmental Parameters

Relative abundances of most PR types and total prokaryotic abundances displayed distinct relationships with environmental variables (**Table 3**). Most inorganic nutrients and cDOM, that showed a strong inshore-offshore gradients along the MacKenzie river plume (Matsuoka et al., 2012; Tremblay et al., 2014) covariated (data not shown) and were negatively correlated with most PR types relative abundances. Such results reflect that high PR concentrations were linked to the nutrient-depleted polar mixed layer. Contributions of the SAR11 PR type followed this general trend and also showed a significant positive correlation with oxygen concentrations (*r* = 0.334, *p* < 0.005). SAR92-related OTU11 was the most notable exception to these prevailing relationships. In contrast to all other PR types, OTU11 abundance increased significantly with salinity, nutrients and chlorophyll concentrations, but decreased with temperature. Four PR types (OTU3a, OTU3b, OTU1 and *Flavobacterium*) decreased significantly with depth. In contrast to the three other types, OTU3a increased significantly with temperature (*r* = 0.366, *p* < 0.005) and light (*r* = 0.266, *p* < 0.05), indicating its preference for coastal epipelagic waters. An inverse relationship was found between relative abundance of *Flavobacterium* PR type and salinity (*r* = -0.302, *p* < 0.05), consistent with the dominance of *Flavobacteria* in bacterial communities of sea-ice and melting water (Staley and Gosink, 1999; Brinkmeyer et al., 2003).

**Table 3:** Pearson’s correlation between environmental parameters and abundance of total bacteria and PR types^a^ over the entire data set.

Variables	Bacteria	SAR11 clade	All PR types	*Alphaproteobacterial PR*				*Gammaproteobacterial PR*		*Flavobacterial PR*	
				SAR11	Arctic group		HOT2C01 OTU 1	SAR92 OTU 11	HTCC2999 OTU 10	*Flavobacterium* sp.
					OTU 3b	OTU 3a				
Depth					-0.452^∗∗∗^	-0.402^∗∗∗^	-0.235^∗^			-0.395^∗∗^
Temperature						0.366^∗∗^		-0.242^∗^		
Salinity								0.287^∗^		-0.302^∗^
O_2_		0.397^∗∗∗^	0.348^∗∗^	0.334^∗∗^			0.246^∗^			
cDOM		-0.390^∗∗^	-0.394^∗∗^	-0.378^∗∗^	-0.424^∗∗∗^		-0.239^∗^		-0.361^∗∗^	-0.274^∗^
PAR	0.263^∗^					0.266^∗^				
NO_3_^2-^		-0.280^∗^	-0.406^∗∗∗^	-0.325^∗∗^	-0.364^∗∗^	-0.324^∗∗^	-0.316^∗^	0.257^∗^		-0.241^∗^
NO_2_^-^	0.247^∗^				-0.314^∗^			0.441^∗∗∗^		-0.296^∗^
NH_4_^+^	0.248^∗^							0.323^∗∗^		
PO_4_^-^					-0.319^∗^	-0.388^∗∗^		0.266^∗^		-0.267^∗^
Si(OH)_4_		-0.395^∗∗^	-0.456^∗∗∗^	-0.423^∗∗∗^	-0.361^∗∗^	-0.273^∗^	-0.286^∗^	0.311^∗^	-0.233^∗^	-0.270^∗^
POC	0.337^∗∗^			-0.280^∗^		0.234^∗^		0.304^∗^		
DOC		-0.261^∗^								
DON		-0.297^∗^	-0.286^∗^	-0.338^∗∗^						
Total Chl*a*	0.466^∗∗∗^			-0.253^∗^				0.569^∗∗∗^		

*^a^Relative abundance of OTU 9 did not correlate with any environmental parameter.*

*^∗^*p* < 0.05; ^∗∗^*p* < 0.005; ^∗∗∗^*p* < 0.0005; Salinity (psu); O_2_: dissolved oxygen (mg.l^-*1*^); cDOM, colored dissolved organic matter (V.m^-*1*^); PAR, photosynthetically active radiation (μmol photons.m^-*2*^.s^-1^); NO_*3*_^2-^, nitrate (μM); NO_*2*_^-^, nitrite (μM); NH_*4*_^*+*^, ammonium (μM); PO_*4*_^-^, phosphate (μM); Si(OH)_*4*_, silica (μM); POC, particulate organic carbon (μM); DOC, dissolved organic carbon (μM); DON, dissolved organic nitrogen (μM); total Chl*a*, total chlorophyll *a* (μg.l^-*1*^).*

## Discussion

Our estimates of PR gene diversity (number of OTUs, S_Chao1_ and H_Shannon_) in the Beaufort Sea waters are similar to that reported in temperate environments (Lami et al., 2009; Riedel et al., 2010). This indicates that low-temperature environments also harbor substantial genetic diversity of PR genes. One half of the PRs retrieved in this study was closely related (>82% amino acid similarity) with sequences widely distributed in low-latitude marine waters. The other half matched with Arctic sequences or belonged to novel OTUs, suggesting that they are endemic. This observation contrasts with previous studies of *puf*M and 16S rRNA gene diversity that revealed larger sets of sequences restricted to Arctic waters (Cottrell and Kirchman, 2009; Ghiglione et al., 2012; Boeuf et al., 2013).

### PR-Based Phototrophy Is the Main Prokaryotic Phototrophic Process in the Arctic Ocean

We observed a substantial overlap between DNA- and cDNA-derived libraries, indicating that a large fraction of the summer PR community actively expressed proteorhodopsin in the Beaufort Sea euphotic waters at the time of sampling. We chose to quantify PR types representing the largest active OTUs by qPCR. Although, the targeted OTUs covered more than half of the recovered sequences, our total numbers are inevitably incomplete. Therefore, they have to be viewed as lower estimates of the actual contribution of PR bacteria to the total bacterial community. These estimates remain lower than those reported from the analysis of the GOS metagenome datasets (about 65%) (Venter et al., 2004; Rusch et al., 2007). The mean abundance values were also lower than previous qPCR analyses that found a PR gene in half of the bacteria in the Sargasso Sea, 23% in the North Atlantic (Campbell et al., 2008), and about 34% in the North Sea (Riedel et al., 2010). However, these sets of data are not easily comparable because the coverage of qPCR primer sets is different between these studies.

Here, we observed that the variability of the measured relative abundances was remarkably high at the PR type level, probably reflecting a heterogeneous distribution of PR bacteria in Arctic waters. This heterogeneity was especially exemplified by SAR11 PR type whose variable proportions were locally higher (up to 45% of the bacterial community) than that found in lower latitude regions (Campbell et al., 2008; Riedel et al., 2010).

Our study demonstrates that contributions of PR-containing organisms outclass those of other phototrophic prokaryotes (cyanobacteria and aerobic anoxygenic phototrophs (AAPs)). Indeed, abundance of cyanobacteria is typically low in marine polar waters (Li et al., 2009) and they were completely absent in Arctic waters of the Chukchi and Beaufort Seas during the Malina cruise (Balzano et al., 2012) as observed previously (Li, 1998). Recently, we reported that AAP bacterial contributions decreased strongly from the Mackenzie River to the open ocean, with a mean of 7% in the Mackenzie mouth, 1% inshore, and 0.1% offshore (Boeuf et al., 2013). Similar proportions (14%) of AAPs and PR-containing bacteria were only obtained in some surface water samples of the Mackenzie plume. We can therefore assume that PR phototrophy is the main prokaryotic phototrophic process in the Beaufort Sea.

### Alphaproteobacterial PR Types are Prevalent in Arctic Waters

According to the qPCR abundance estimates, alphaproteobacterial PR genes represented about 95% of the PR community in all sampled waters. This result could appear surprising because estimates of gene abundance based on frequencies in clone libraries were almost similar for alphaproteobacterial and gammaproteobacterial sequences. By inspecting the sequence templates of the PCR primer set in the limited number of strains whose genome is available yet, we found that preferential amplification of gammaproteobacterial PR sequences may explain these discrepancies. However, although *Gammaproteobacteria* were overestimated in our PR clone libraries, 16S rRNA pyrosequencing analyses demonstrated that they represent one of the two most abundant bacterial groups in the Arctic Ocean, accounting for about 20–30% of the total bacterial community (Kirchman et al., 2010; Comeau et al., 2011).

Although SAR11 PR type was moderately abundant in clone libraries, qPCR estimates revealed it as the dominant clade in coastal and oceanic waters of the Beaufort Sea. Interestingly, its proportions in Arctic oceanic waters were close to those measured in the North Atlantic (Campbell et al., 2008) and higher than those found in the North Sea (Riedel et al., 2010), two marine environments where other alphaproteobacterial PR genes dominated. In most samples other than surface, we found that SAR11 PR type made up at least for 70% of the PR arctic community, revealing a remarkable adaptability to summer arctic conditions.

OTU3a, the second most abundant PR type, was previously reported in coastal waters of the Chukchi and western Beaufort Seas (Cottrell and Kirchman, 2009). Together with our data, the latter study suggests that this clade is restricted to high latitudes. Our quantitative estimates indicated that it made up similar proportions than of SAR11 PR type in several stations in the coastal Beaufort Sea and even outgrew it in the productive waters of the Chukchi Sea. Little is known about this clade but our results suggest that it could transiently represent a prominent and active component of the bacterioplankton in surface Arctic waters.

### Proteorhodopsin in the Arctic SAR11 Community

To evaluate PR gene contribution within the SAR11 community, we assessed the relative abundance of SAR11 16S rRNA gene copies by qPCR. Our estimates of SAR11 contributions (about 30% of the total prokaryotes) were in agreement with CARD-FISH experiments (Malmström et al., 2007; Alonso-Sáez et al., 2008) and 454 sequencing analysis (Comeau et al., 2011) conducted in Arctic waters. Based on our average estimates obtained by qPCR, only about a third of SAR11 bacteria contained a PR gene. However, proportions of SAR11 containing the PR gene in the SAR11 community were highly heterogeneous, ranging from 5 to 90% according locations. This result contrasts with previous data from field experiments that found proteorhodopsin in about 80% of the SAR11 community (Campbell et al., 2008) and from comparative analysis of seven genomes from different SAR11 subclades that revealed one PR gene per genome (Grote et al., 2012). These data suggest that PR phototrophy is likely to be present in only some SAR11 populations and could be in line with the observation of a differential distribution patterns of SAR11 subclades or ecotypes (Field, 1997; Brown and Fuhrman, 2005), which follows a strong zonation according latitude or depth (Brown et al., 2012). Interestingly, the dominant SAR11 phylotype (P1a) in polar waters contained numerous PR-containing strains (Brown et al., 2012). In our study, the fact that the PR gene was not homogeneously found in the SAR11 community could suggest a distribution according ecotypes rather than phylotypes or at finer phylogenetic levels than previously defined.

### Contextual Parameters Explaining PR Community Structure

Studies of PR gene expression in natural waters as well as in light–dark incubations of model bacteria in culture demonstrated that the PR gene can be strongly upregulated by light (Lami et al., 2009) and by nutrient limitation (Akram et al., 2013). Environmental conditions in the Arctic Ocean range from constant light and nutrient depletion during summer to complete darkness and sea ice during winter. In the Beaufort Sea, the abundance of most PR types increased with decreasing nutrient and cDOM concentrations while the abundance of only one PR type was positively correlated with light. The pelagic Beaufort Sea is oligotrophic during summer as a result of the strong vertical stratification caused by Mackenzie River inputs and sea-ice melting (Carmack and MacDonald, 2002). These low-nutrient conditions prevailed during the Malina cruise where low surface nitrate concentrations limiting heterotrophic bacterial growth were observed (Ortega-Retuerta et al., 2012). Our data suggest that in the arctic conditions, adaptation to nutrient limitation rather than light availability would regulate the abundance and distribution of PR bacteria. This is consistent with the presence of PR-containing bacteria in both summer and winter (Cottrell and Kirchman, 2009; Nguyen et al., 2015) and their activity in the ice at low light intensities (Koh et al., 2010).

Summer oligotrophic conditions as inducing factors of PR gene expression would be also consistent with the high proportions of active PR bacteria we found in the Beaufort Sea. It is conceivable that energy gained by phototrophy may confer to PR bacteria the capacity to better face the low-nutrient conditions. This hypothesis is supported by the observation that some PR bacteria better survive low-nutrient conditions (Gómez-Consarnau et al., 2010; Steindler et al., 2011). We found that the active PR community was dominated by SAR11 members, whose abundance was negatively correlated with Chl *a*, POC and DON concentrations. These data reinforce the idea that SAR11 is well-adapted to nutrient-limited oligotrophic marine environments (Malmström et al., 2005; Lauro et al., 2009). However, the dominance and activity of SAR11 PR type within the large environmental gradients of the Beaufort Sea indicate that their unique metabolic capacities allow them to thrive well in a wide range of trophic conditions, challenging the notion that these oligotrophs are adapted for the efficient use of dissolved substrates (Giovannoni et al., 2005).

By contrast to other PR types, the relative abundance of SAR92-related PR (OTU11) increased along the nutrient, cDOM and Chl*a* concentrations. Such an observation indicates that these organisms are competitive at high organic matter and inorganic nutrient concentrations supplied during the high-discharge period of the MacKenzie River. This idea is consistent with the observation that SAR92 clade members are responsive to phytoplankton blooms induced by inorganic nutrient enrichment in microcosm experiments (Pinhassi et al., 2005) and aided the isolation of SAR 92 strains from the nutrient-rich upwelling off the Oregon coast (Stingl et al., 2007a). An active role in the breakdown of algae-derived compounds has been recently suggested for these organisms (Teeling et al., 2012). Although the growth of HTCC2207, the first cultivated SAR92 strain, was found to be limited by the amount of available carbon (Stingl et al., 2007a), SAR92 members may be adapted to more nutrient-rich patches in the natural environment and responds more readily to phytoplankton bloom dynamics. First physiological analyses revealed that HTCC2207 showed no responses to light (Stingl et al., 2007a) and the benefit of PR phototrophy for this organism has not been identified yet.

In summer, ice melt-induced stratification of the water column, local and transient phytoplankton blooms as well as aggregation of particulate organic matter contribute to the seascape variability in the Beaufort Sea heavily impacted by the Mackenzie River and smaller rivers. Moreover, numerous biotic processes result in patchy resource distributions. The different responses of PR types to environmental parameters mirror the strong spatial variability of their abundance. Together, our results suggest that PR phototrophy may help microorganisms thriving in extreme arctic conditions and favor energy production to exploit this heterogeneity during the Arctic summer.

## Conclusion

This diversity and qPCR analyses of PR sequence genes in marine waters of the Arctic Ocean generated novel insights into the composition and spatial distribution of PR-containing bacteria. The PR amplicons showed great spatial divergence in composition, and were frequently dominated by genes related to taxonomically diverse organisms, most of them being expressed at the time of sampling. Although, the abundance of PR-containing bacteria is highly variable between locations, depths and taxonomic clades, our results indicate that PR phototrophy may be a significant process contributing to the carbon cycle in the Arctic Ocean.

The ecological mechanisms that underlie this variability and the energetic benefits of PR-based phototrophy in the world’s oceans remains to be elucidated.

## Author Contributions

All authors were substantially involved in the data acquisition and/or data analyses. DB and CJ wrote the first draft of the manuscript and all authors contributed substantially to revisions.

## Conflict of Interest Statement

The authors declare that the research was conducted in the absence of any commercial or financial relationships that could be construed as a potential conflict of interest.
