# Supplementary material for: Summer Abundance and Distribution of Proteorhodopsin Genes in the Western Arctic Ocean
Source: Front Microbiol. 2016 Oct 13;7:1584. doi: 10.3389/fmicb.2016.01584 (PMC5061748; doi:10.3389/fmicb.2016.01584)
Supplement: Supplementary file 1 [file Data_Sheet_1.PDF]

Supplementary Information

Collection of ancillary data

High vertical resolution environmental data were collected between 0 and up to 80 m depth using a conductivity-temperature-depth-oxygen profiler (CTDO, Seabird 911 plus) with a rosette supporting 24 12 L Niskin bottles equipped with silicon rubber closures. Inorganic nutrients (nitrate, nitrite, phosphate and silicate) and organic constituents [particulate organic carbon (POC), dissolved organic carbon (DOC) and dissolved organic nitrogen (DON)] were measured as detailed in Tremblay et al. (2014). Chlorophyll *a* concentrations were determined by HPLC, following the method described in (Ras et al., 2008). CDOM fluorescence and PAR was obtained using a Backscat 1 fluorometer (Dr. Haardt Optik-Mikroelektonik) and a sensor (Biospherical Instruments), respectively, mounted on the rosette. Temperature, salinity, cDOM and PAR data used in the analyses were mean values of data collected between -0.5 m and +0.5 m of each sampled depth. Other parameters were measured from samples collected from the Niskin bottles mounted on the rosette.

Isolation and identification of arctic PR-containing bacteria

Seawater samples (50-100 µl) collected at the surface and at the deep chlorophyll maximum (DCM) were spread onto low-nutrient heterotrophic medium (Cho and Giovannoni, 2004) prepared using seawater collected at station 860 at the surface and the DCM, respectively. Dimethylsulfoniopropionate (DMSP) was added to the medium at a final concentration of 100 nM (Stingl et al., 2007). Culture plates were incubated for up to 2 months at 4°C into thermostatic cabinets illumined by luminescent tubes at irradiance levels of 120 µmol photons.m^{-2}.s^{-1} (for surface samples) and 25 µmol photons.m^{-2}.s^{-1} (for DCM samples) under 10:14 light-dark cycle to mimic light conditions at the time of sampling.

Bacterial DNA was extracted using the QuickExtract™ Bacterial DNA Extraction Kit (Epicentre Biotechnologies, Madison, WI, USA*)*. PR-containing isolates were screened by terminal restriction fragment length polymorphism (T-RFLP) of 16S rRNA genes, following protocols previously described in Boeuf et al. (2013). PR and 16S rRNA gene sequences of at least two randomly chosen strains from each T-RFLP group (if applicable) were amplified by PCR (Table S1). When necessary, PCR products were gel-purified using the DNA gel extraction kit (Millipore, Billerica, MA, USA) and sequenced with an ABI 3130 POP7 sequencer (Applied Biosystems).

All 16S rRNA gene sequences were compared to sequences available in public databases with BLASTn (Altschul et al., 1997). The new sequences were aligned according the secondary structure of the rRNA using the Q-INS-I strategy from MAFFT sequence alignment program version 6.5 (Katoh et al., 2009). Phylogenetic affiliations of strains were computed using Bayesian, neighbor-joining, and maximum likelihood inference approaches using MrBayes (Ronquist and Huelsenbeck, 2003) and MEGA 5 softwares (Tamura et al., 2011), respectively. The 16S rRNA and PR gene sequences of the isolates are available in GenBank database under the accession numbers JX863350 to JX863365 and JX863334 to JX863349, respectively.

Diversity of PR-containing isolates

To gain more insights into the phylogenetic affiliation of the PR genes obtained in this study, PR-containing bacteria were isolated from Beaufort Sea samples. Using the PR-1aF and PR-1aR primer set (Table S1), PR genes were detected in 25 of 470 isolates tested. Diverse PR-containing isolates were obtained from offshore and shelf waters of the Beaufort Sea. Based on T-RFLP analyses of 16S rRNA gene, they were distributed in 7 groups (Table S4). The major T-RFLP group (11 strains) was affiliated with the gammaproteobacterial genus

Glaciecola and the other six with the family *Flavobacteriaceae*. Among *Flavobacteriaceae*, isolated strains belonged to the genera *Flavobacterium*, *Psychroflexus*, *Polaribacter* and *Maribacter* (data not shown). 16S rRNA genes of all the isolates were most closely related with those of strains or environmental clones retrieved from polar seawater or sea ice samples (Table S4). PR sequences of some isolates grouped with OTUs retrieved in this study. *Glaciecola* isolates had PR amino acid sequences highly similar to that of OTU 20 (> 84% identity) (Fig. 1) whereas PR sequences of *Polaribacter* strains RCC2386 and RCC2387 were highly similar (> 88% identity) to that of OTU52. Interestingly, strain RCC2386 and an environmental clone obtained from the Chukchi Sea (Cottrell and Kirchman, 2009) has identical PR. Other isolates did not fall in any OTU obtained in this study.

Light-tuned PR type distribution

A large majority (81%) of the predicted PR proteins retrieved in this study contained leucine or methionine at position 105, suggesting that they are green-absorbing PRs (Fig. S5). Green-absorbing PRs were dominant in all surface samples. Among them, all PR sequences that carried a methionine at this position belonged to *Bacteroidetes*. They were present only in surface libraries and their relative abundance was higher in river-influenced stations. Relative abundances of blue-absorbing PRs, with a glutamine at position 105, increased in offshore surface stations of the North Pacific Ocean and the Beaufort Sea. They also increased with depth and dominated at depth at station 170 where an upwelling probably occurred.

Table S1. Summary of primers used in this study.

Experiment	Gene	Group	Primer name	Primer sequence (5'-3')	Annealing temp (°C)	Amplicon length (bp)	Reference
t-RFLP	*16S*	*Bacteria*	27F<br>1492R	AGAGTTTGATCMTGGCTCAG<br>CGGTTACCTTGTTACGACTT	55	1465	(Lane, 1991)<br>(Weisburg et al., 1991)
PCR	*16S*	*Bacteria*	8F<br>1492R	AGAGTTTGATCCTGGCTCAG<br>CGGTTACCTTGTTACGACTT	55	1465	(Edwards et al., 1989)<br>(Weisburg et al., 1991)
RT/PCR	*PR*	PR-containing bacteria	PR-1aF<br>PR-1aR	GATCGAGCGNTAYRTHGA**Y**TGG*<br>GATCGAGCRTADATNGCCCANCC	55	345	(Campbell et al., 2008)<br>(Campbell et al., 2008)
qPCR	*16S*	*Bacteria*	BACT1369F<br>PROK1492R	CGGTGAATACGTTCYCGG<br>GGWTACCTTGTTACGACTT	56	142	(Suzuki et al., 2000)<br>(Suzuki et al., 2000)
qPCR	*16S*	SAR 11	433F<br>588R	CTCTTTCGTCGGGGAAGAAA<br>CCACCTACGWGCTCTTTAAGC	59	155	(Suzuki et al., 2001)<br>(Suzuki et al., 2001)
qPCR	*PR*	SAR 11	125F<br>288R	THGGWGGATAYTTAGGWGAAGC<br>CCCAACCWAYWGTWACRATCATTCT	56	183	(Campbell et al., 2008)<br>(Campbell et al., 2008)
qPCR	*PR*	OTU 3a	PRS472F<br>PRS567R	ATCGTAGGCATGGTAGGC<br>CTTGACAGATTCTGGAGC	55	95	(Cottrell and Kirchman, 2009)<br>(Cottrell and Kirchman, 2009)
qPCR	*PR*	OTU 3b	OTU3b-a14f<br>OTU3b-b2r	CCTTCGTGCGATTACTGC<br>CCAAGCGCTGATGTAGCC	57	112	This study<br>This study
qPCR	*PR*	OTU 11	OTU11-f6<br>OTU11-r2	CTGTGCCGCTGCTAATGA<br>TCCCAGATAATGAAGCCC	58	193	This study<br>This study
qPCR	*PR*	OTU 9	OTU9-a1f<br>OTU9-b14r	GGTGTGTTTTGGCGTTTG<br>CTGCACATTGGCGTTTCC	62	189	This study<br>This study
qPCR	*PR*	OTU 1	OTU1-a16f<br>OTU1-b27r	GGAATGGCAGCTTGGTTA<br>AAAGCAGTTTGTGCTGGG	48	98	This study<br>This study
qPCR	*PR*	OTU 10	OTU10-f3<br>OTU10-r21	CCACTGCAGATTGTCGAG<br>GATATACAACCAACCGGC	56	180	This study<br>This study
qPCR	*PR*	*Flavobact-erium* sp.	Flavo-f6<br>Flavo-r2	GGGCTAAGCAGTCTTTGT<br>TGTGTGCAGCAAGTGTGT	60	206	This study<br>This study

* a typographical error exists in the original publication (R instead of bolded Y)

Table S2. Clone library comparison of seawater samples collected during the MALINA cruise in summer 2009.

Station[a]	Depth (m)	Template type	Nb. of clones	Nb. of OTUs[b]	Coverage (%)	S_{Chao1} (LCI-UCI)[c]	$H_{Shannon}$ (LCI-UCI)
PAC2	0m	DNA	32	9	94%	9.3 (9-14)	1.99 (1.75-2.22)
ARC1	0m	DNA	30	8	90%	9 (8-19)	1.58 (1.21-1.95)
170	3m	DNA	30	8	93%	8.3 (8-14)	1.83 (1.57-2.09)
	3m	cDNA	12	3	92%	3 (3-NA[d])	0.72 (0.26-1.19)
	20m	cDNA	26	3	100%	3 (3-3)	0.69 (0.37-1)
320	3m	cDNA	39	10	85%	15 (11-39)	1.59 (1.24-1.95)
360	3m	DNA	31	8	87%	14 (9-46)	1.61 (1.27-1.95)
	3m	cDNA	39	12	90%	13.5 (12-24)	2.19 (1.93-2.45)
	50m	cDNA	36	6	100%	6 (6-6)	1.67 (1.5-1.84)
	50m	DNA	32	9	91%	10 (9-20)	1.83 (1.52-2.14)
398	0m	DNA	39	19	62%	54 (29-145)	2.37 (1.97-2.77)
620	3m	DNA	15	8	80%	8.6 (8-15)	1.97 (1.63-2.31)
	3m	cDNA	38	15	84%	17.5 (15-30)	2.35 (2.03-2.67)
	65m	cDNA	33	2	100%	2 (2-2)	0.23 (0-0.46)
	65m	DNA	28	7	93%	7.3 (7-13)	1.58 (1.26-1.9)
680	3m	cDNA	42	17	74%	35.3 (21-92)	2.36 (2.04-2.69)
	40m	cDNA	46	10	89%	20 (12-62)	1.83 (1.57-2.09)
	40m	DNA	9	6	56%	9 (6-29)	1.68 (1.21-2.15)
694	0m	DNA	60	24	73%	48 (31-105)	2.42 (2.05-2.78)

[a] Exact locations of the stations are shown in Fig. S1.
[b] Operational taxonomic units (OTUs) were defined with MOTHUR assuming a 82% amino acid sequence similarity level.
[c] LCI and UCI are the lower and upper bound of 95% confidence interval of the Chao1 and Shannon indexes, respectively.
[d] NA, not applicable

Table S3. Relative abundances of PR sequence types (%) as quantified by qPCR in surface seawater samples collected along the transect from Vancouver to the Beaufort Sea. The estimates were normalized to 16S rRNA gene abundance.

**Affiliation**	**Group targeted**	**Stations**[a]					
		**PAC1**	**PAC2**	**BER**	**ARC1**	**ARC2**	**BEA1**
Alphaproteobacteria							
	SAR11	7.1	7.8	3.7	2.7	0.6	4.3
Arctic clade	OTU3a	-	2.4	0.4	36.7	3.8	1.2
Arctic clade	OTU3b	0.1	0.3	0.5	1.4	0.1	0.5
HOT2C01-related	OTU1	4.9	2.2	0.3	2.1	0.7	2.9
Alphaproteobacteria	Total	**12.1**	**12.7**	**4.9**	**42.9**	**5.2**	**8.8**
Gammaproteobacteria							
SAR92	OTU9	<0.1	0	0.4	0.7	<0.1	<0.1
SAR92	OTU11	<0.1	<0.1	<0.1	0.1	0.14	<0.1
HTCC2999-related	OTU10	<0.1	0.1	<0.1	0.3	<0.1	0.11
Gammaproteobacteria	Total	**<0.1**	**0.2**	**0.4**	**1.2**	**0.3**	**0.2**
Flavobacteria	*Flavobacterium* sp.	0	0	0	0	1.9	<0.1
	**Total**	**12.2**	**12.8**	**5.3**	**44.1**	**7.4**	**9.1**

[a] Exact locations of the stations are shown in Fig. S1.

Table S4. Main features of T-RFLP groups determined among 25 arctic PR-containing isolates and affiliation of their representative strains.

T-RF size (bp)		Nb of strains	Representative strains	16S rRNA gene closest relative				PR closest relative			
FAM[a]	HEX[b]			Name	Accession number	Similarity (%)	Isolation site	Name	Accession number	Similarity (%)	Isolation site
84	112, 115, 127	7	RCC 2392 (RCC 2393, 2394, 2396, 2399)	strain ARK10272	AF468432	99.9	Arctic Ocean	*Flavobacteria* bacterium BAL38	ZP_01734914	83.7	Baltic Sea
83	112, 115, 127	2	RCC 2397	*Flavobacterium* sp. R-38477	FR772059	98.9	Antarctica	*Flavobacterium frigoris* PS1	ZP_09895557	77.6	Ross Sea
325	20, 121, 165, 181	2	RCC 2388 (RCC 2391)	*Psychroflexus* sp. gap-d-25	DQ530461	99.7	Antarctic sea ice	*Psychroflexus torquis* ATCC700755	ZP_01253360	86.2	Southern Ocean
87, 325	112, 115, 127	1	RCC 2386	*Polaribacter* sp. BSw20011	EU365593	100	Arctic Ocean	Clone PR_108_08_01	ACM79430	100	Arctic Ocean
87	112, 115, 127	1	RCC 2387	Fosmid ARCTIC40_B_04	EU795091	100	Arctic Ocean	Uncultured sea ice bacterium	ADB27806	82.2	Ross Sea
86, 89	77, 127	1	RCC 2395	Strain ARK10223	AF468428	100	Arctic Ocean	Clone 1812216	ABY66923	81.6	Indian Ocean
329	20, 120, 165	11	RCC 2389 (RCC 2390, 2398)	Clone DFCb33	HQ230172	100	Arctic snow	*Glaciecola nitratireducens* FR1064	YP_004870549	87.9	East China Sea

[a]FAM, carboxyfluorescein; [b]HEX, hexachlorofluorescein; T-RF, terminal restriction fragment; RCC, Roscoff Culture Collection

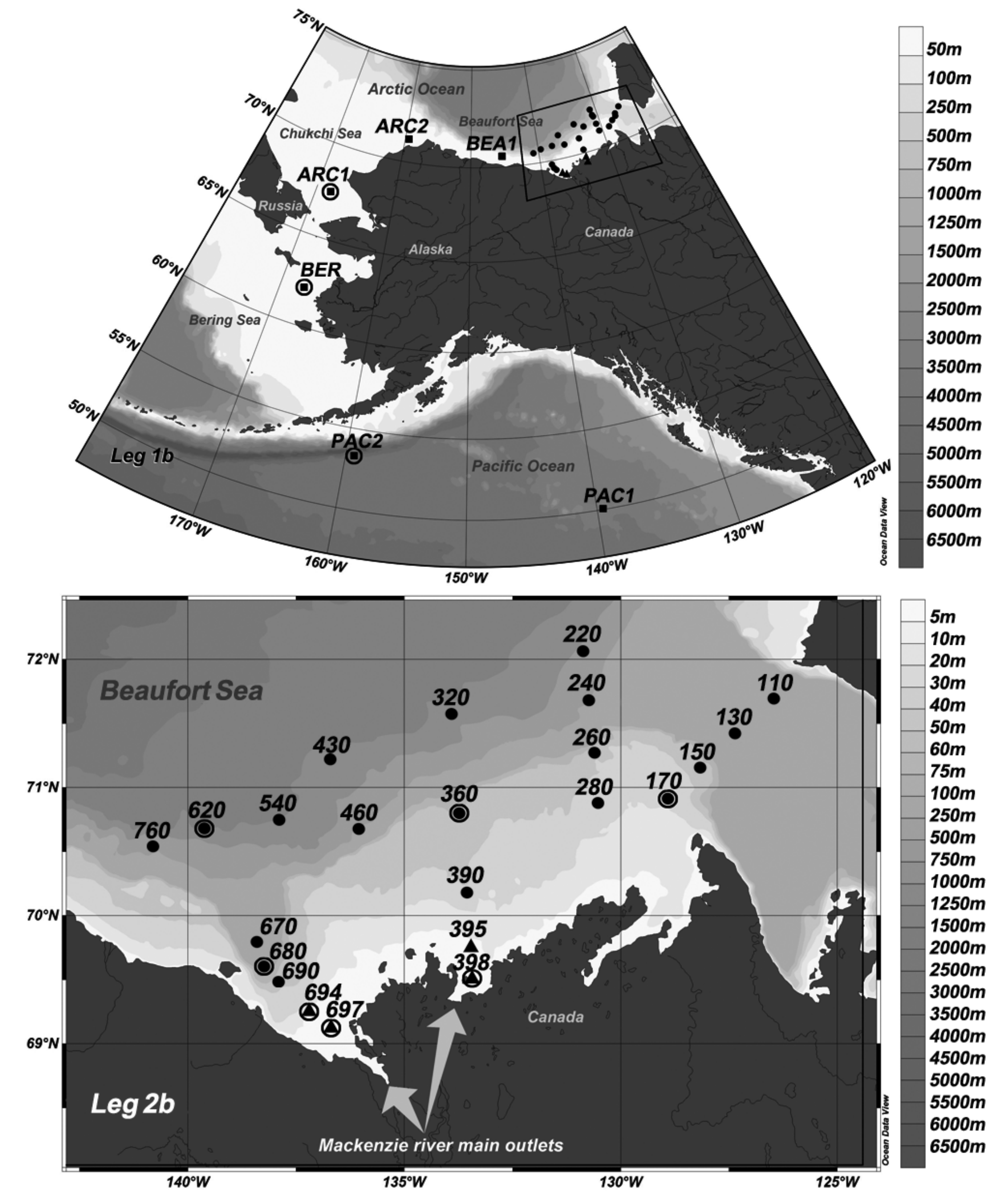

Fig. S1. Locations of stations sampled during legs 1b and 2b of MALINA cruise. Leg 1b stations (black squares) were sampled only at the surface; leg 2b stations were sampled at several depths (black dots) or only at the surface (black triangles). Circles indicate stations where PR gene libraries were constructed. Grey shades correspond to bottom depths.

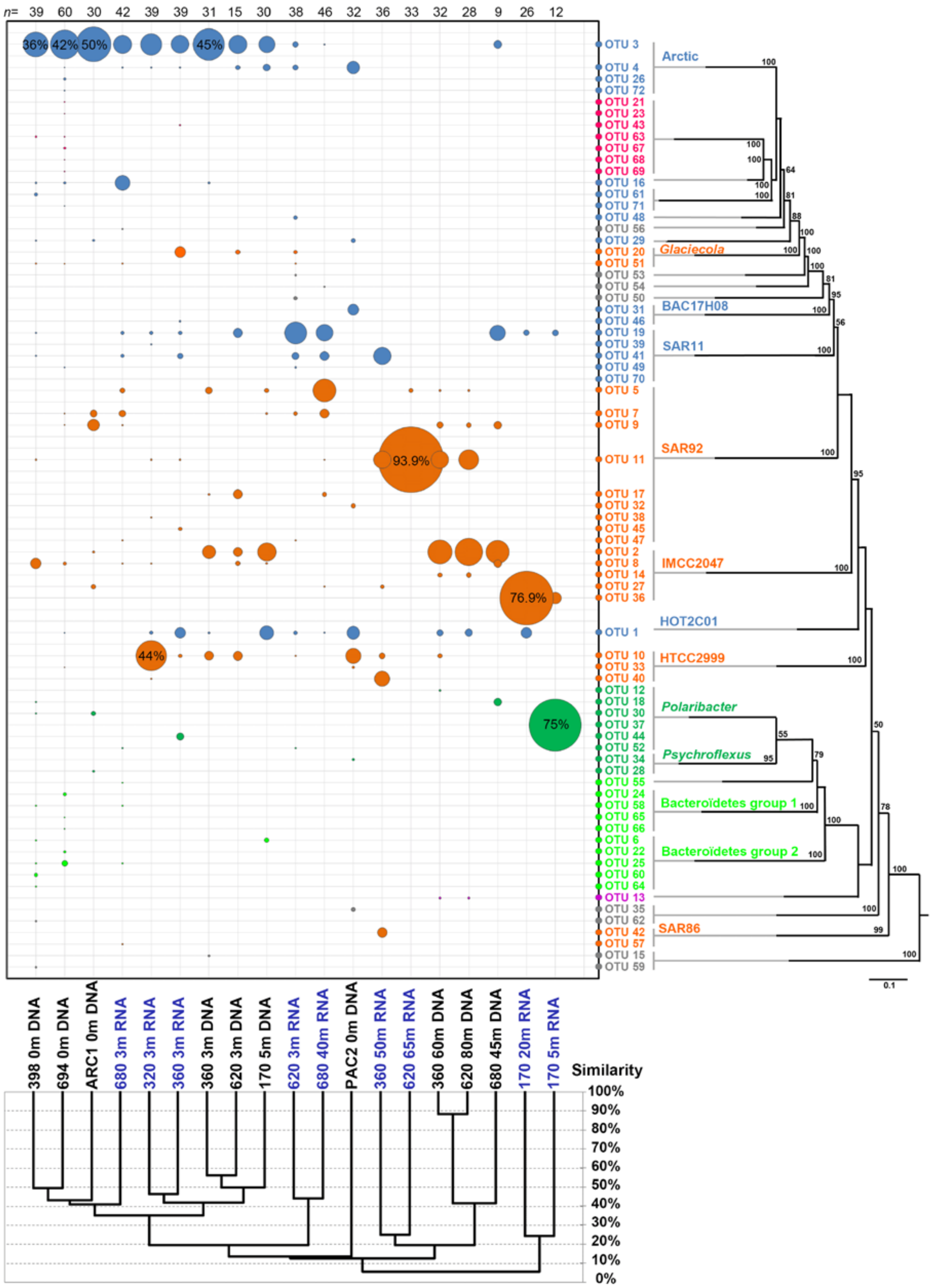

Fig. S2. Relative abundance of OTUs in clone libraries constructed from DNA (in black) or cDNA (in blue). The dendrogram (bottom) represents the similarity between the PR assemblages in the gene libraries. Numbers of clones (*n*) per library is indicated. The relationships between OTUs are shown in the phylogenetic tree according to Fig. 1 (the scale bar represents the number of amino acid substitution per site). OTUs are colored according to phylogenetic affiliation (*Alphaproteobacteria* in blue, *Gammaproteobacteria* in orange and *Bacteroidetes* in green).

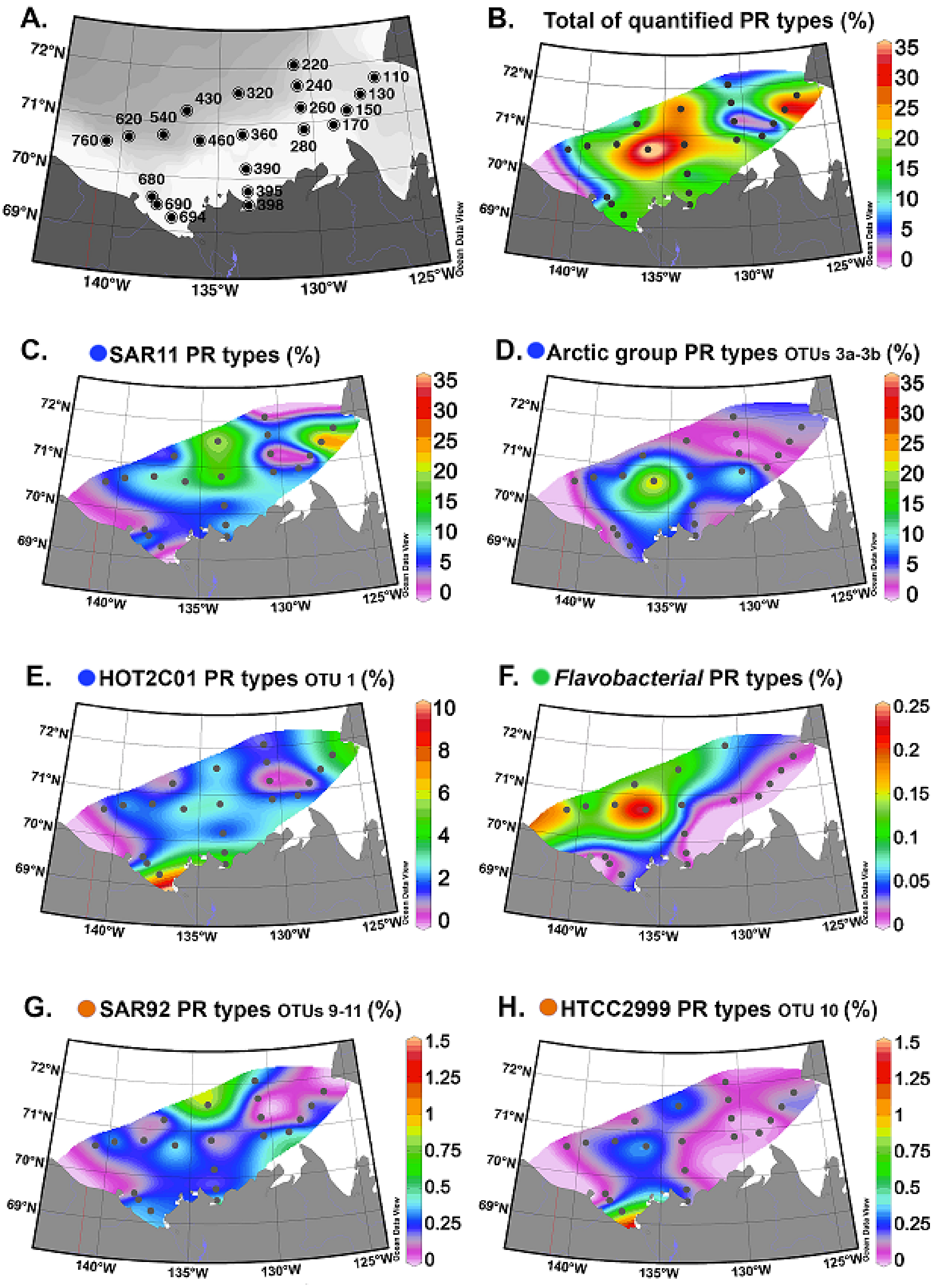

Fig. S3. Surface distributions of major PR types in the coastal Beaufort Sea during July and August 2009.

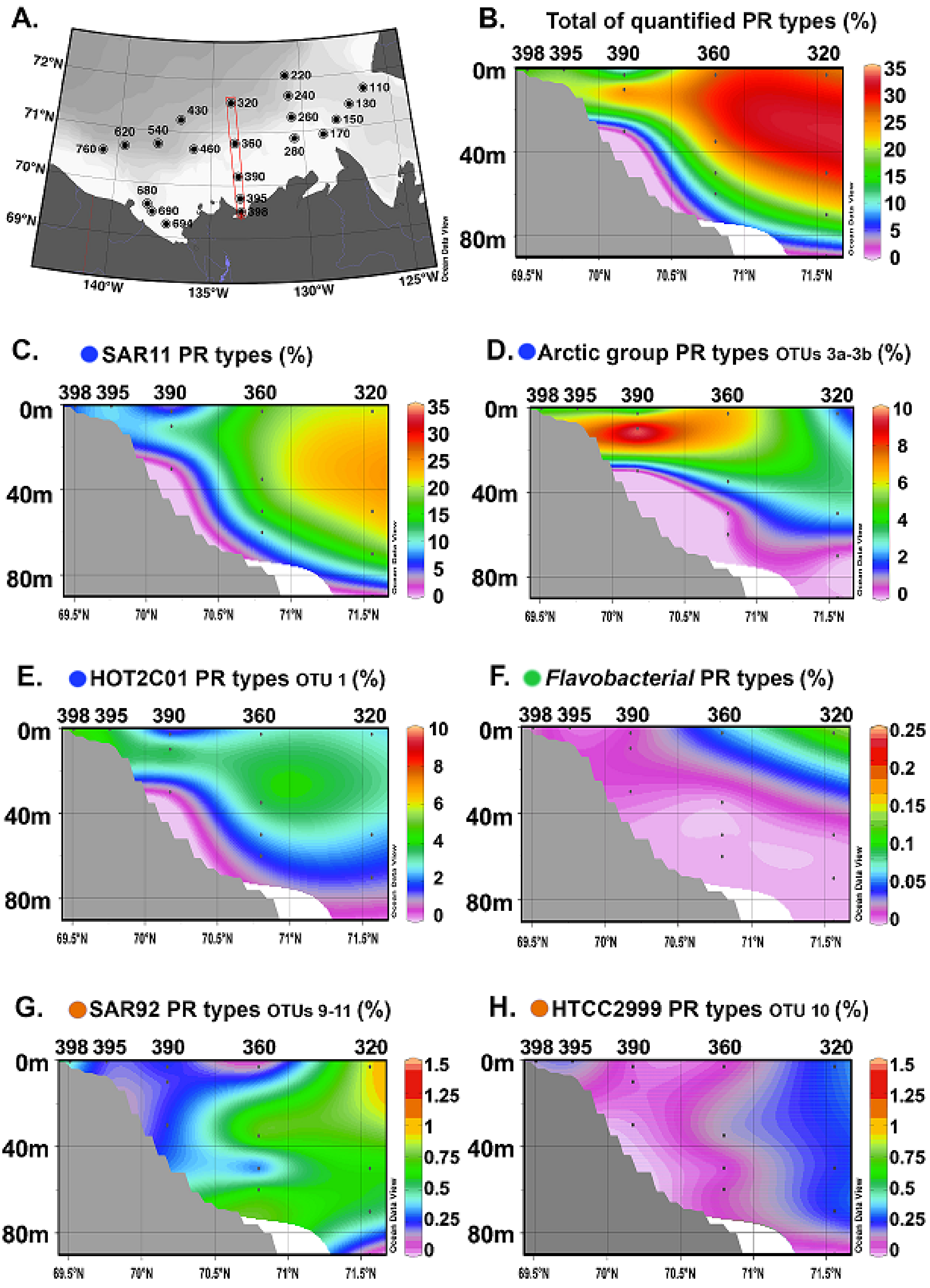

Fig. S4. Vertical distribution of major PR types along transect 300 in the Beaufort Sea during July and August 2009.

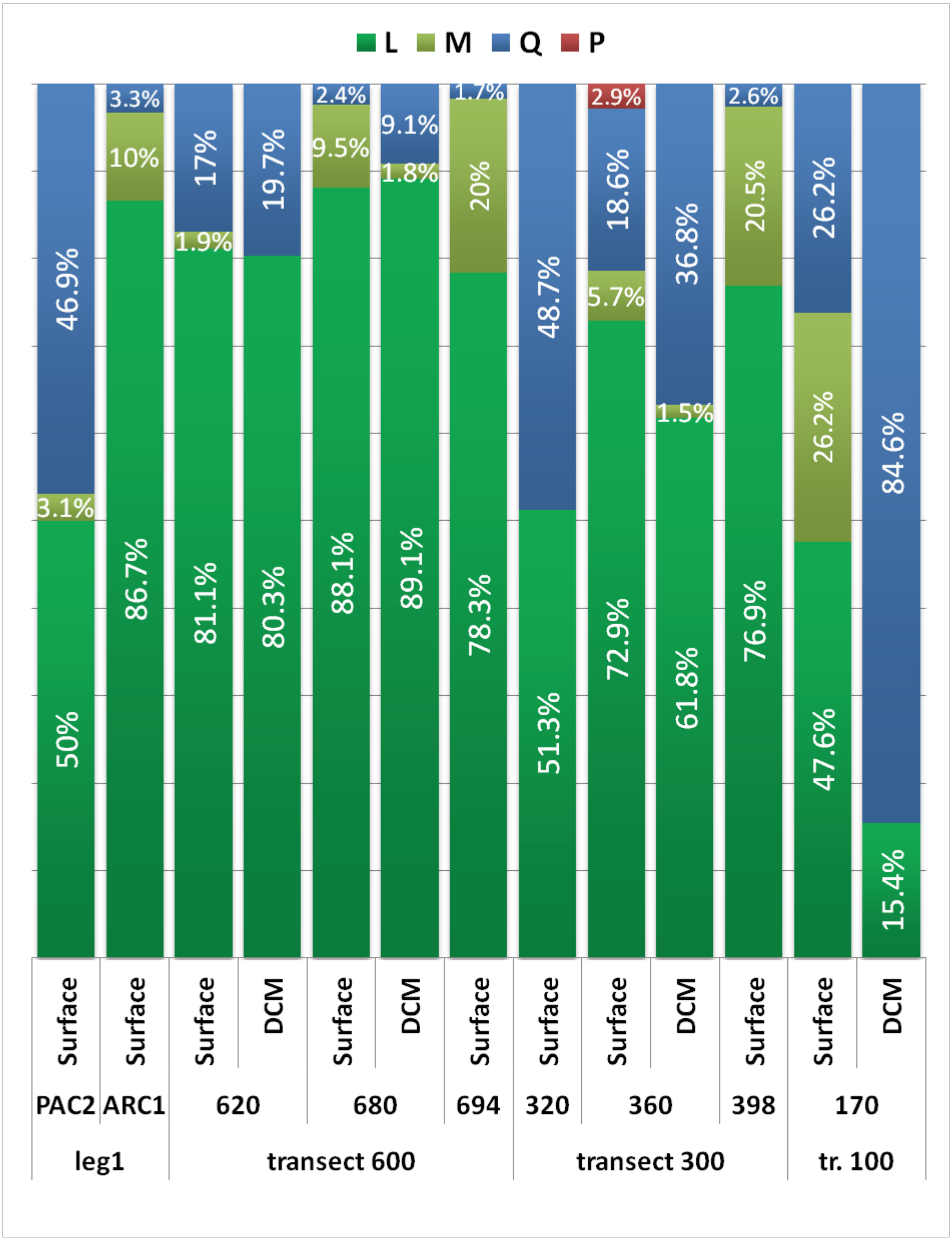

Fig. S5. Relative abundance in clone libraries of PR spectral variants along transects surveyed during the Malina cruise (Fig. S1). Green-absorbing variants with leucine (L) or methionine (M) at position 105 are indicated in green; blue-absorbing variants with glutamine (Q) at position 105 are indicated in blue; variants with proline (P) at position 105 are indicated in red and they have not been yet characterized.

References

Altschul, S.F., Madden, T.L., Schäffer, A.A., Zhang, J., Zhang, Z., Miller, W., et al. (1997). Gapped BLAST and PSI-BLAST: a new generation of protein database search programs. *Nucleic Acids Res.* 25(17), 3389-3402.

Boeuf, D., Cottrell, M.T., Kirchman, D.L., Lebaron, P., and Jeanthon, C. (2013). Summer community structure of aerobic anoxygenic phototrophic bacteria in the western Arctic Ocean. *FEMS Microbiol. Ecol.* 85(3), 417-432. doi: 10.1111/1574-6941.12130.

Campbell, B.J., Waidner, L.A., Cottrell, M.T., and Kirchman, D.L. (2008). Abundant proteorhodopsin genes in the North Atlantic Ocean. *Environ. Microbiol.* 10(1), 99-109.

Cho, J.C., and Giovannoni, S.J. (2004). Cultivation and growth characteristics of a diverse group of oligotrophic marine Gammaproteobacteria. *Appl. Environ. Microbiol.* 70(1), 432-440. doi: http://dx.doi.org/10.1128/AEM.70.1.432-440.2004

Cottrell, M.T., and Kirchman, D.L. (2009). Photoheterotrophic microbes in the Arctic Ocean in summer and winter. *Appl. Environ. Microbiol.* 75(15), 4958-4966.

Edwards, U., Rogall, T., Blöcker, H., Emde, M., and Böttger, E.C. (1989). Isolation and direct complete nucleotide determination of entire genes. Characterization of a gene coding for 16S ribosomal RNA. *Nucleic Acids Res.* 17(19), 7843-7853.

Katoh, K., Asimenos, G., and Toh, H. (2009). Multiple alignment of DNA sequences with MAFFT. *Methods Mol. Biol.* 537, 39-64.

Lane, D.J. (1991). *Nucleic acid techniques in bacterial systematics.* John Wiley & Sons.

Ras, J., Claustre, H., and Uitz, J. (2008). Spatial variability of phytoplankton pigment distributions in the Subtropical South Pacific Ocean: comparison between in situ and predicted data. *Biogeosciences* 5(2), 353-369. doi: 10.5194/bg-5-353-2008.

Ronquist, F., and Huelsenbeck, J.P. (2003). MrBayes 3: Bayesian phylogenetic inference under mixed models. *Bioinformatics* 19(12), 1572-1574. doi: http://doi.org/10.1093/bioinformatics/btg180.

Stingl, U., Tripp, H.J., and Giovannoni, S.J. (2007). Improvements of high-throughput culturing yielded novel SAR11 strains and other abundant marine bacteria from the Oregon coast and the Bermuda Atlantic Time Series study site. *ISME J.* 1(4), 361-371.

Suzuki, M.T., Preston, C.M., Chavez, F.P., and DeLong, E.F. (2001). Quantitative mapping of bacterioplankton populations in seawater: field tests across an upwelling plume in Monterey Bay. *Aquat. Microb. Ecol.* 24(2), 117-127.

Suzuki, M.T., Taylor, L.T., and DeLong, E.F. (2000). Quantitative analysis of small-subunit rRNA genes in mixed microbial populations via 5'-nuclease assays. *Appl. Environ. Microbiol.* 66(11), 4605-4614.

Tamura, K., Peterson, D., Peterson, N., Stecher, G., Nei, M., and Kumar, S. (2011). MEGA5: Molecular Evolutionary Genetics Analysis using Maximum Likelihood, Evolutionary Distance, and Maximum Parsimony Methods. *Mol. Biol. Evol.* 28(10), 2731-2739.

Weisburg, W.G., Barns, S.M., Pelletier, D.A., and Lane, D.J. (1991). 16S ribosomal DNA amplification for phylogenetic study. *J. Bacteriol.* 173(2), 697-703.
